# From perceptions to beliefs: the mediating role of attitudes in pre-service English teacher cognition of Communicative Language Teaching in China

**DOI:** 10.3389/fpsyg.2026.1736617

**Published:** 2026-01-20

**Authors:** Jingyao Zhou, Dong Liu

**Affiliations:** 1 School of Foreign Languages, Zhengzhou Normal University, Zhengzhou, China; 2 School of Business Administration, Shandong Women’s University, Jinan, China

**Keywords:** belief–practice gap, China, Communicative Language Teaching, pre-service teachers, teacher cognition

## Abstract

**Introduction:**

Although Communicative Language Teaching (CLT) has gained global endorsement, its adoption in exam-oriented EFL contexts like China is inconsistent. While prior studies emphasize in-service teachers, limited research explores pre-service teachers, whose cognitions will influence future language pedagogy. This study investigates how pre-service English teachers’ perceptions of secondary school classrooms shape their attitudes toward grammar- versus communication-focused instruction, and subsequently their beliefs about language learning, with attitudes as a mediator. It also examines how this interrelation differs by gender and English learning starting age.

**Methods:**

Based on a large-scale survey (*n* = 865) of English majors in China, data were analyzed via descriptive statistics, *t*-tests, exploratory and confirmatory factor analyses, and structural equation modeling (SEM).

**Results:**

Findings reveal dominant grammar-focused perceptions, yet stronger attitudes favoring communicative approaches. SEM results confirm perceptions indirectly influence beliefs through attitudes, underscoring attitudes’ mediating role in teacher cognition. Subgroup analyses show variations, with females and early starters exhibiting stronger communicative orientations.

**Discussion:**

These insights highlight the enduring belief-practice gap in high-stakes environments and advocate for teacher training, curriculum reforms, localized CLT adaptations, and AI integration (such as through personalized simulations and real-time feedback) to foster communicative practices and bridge preparation deficiencies.

## 1 Introduction

Over the past four decades, Communicative Language Teaching (CLT) has emerged as a dominant paradigm in English language education, championed for its role in developing learners’ communicative competence in authentic contexts (Richards and Rodgers, 2014; Larsen-Freeman and Anderson, 2011). In contrast to traditional grammar-translation and audiolingual approaches, CLT prioritizes meaning negotiation, interactive tasks, and real-world language use through activities like role plays and discussions (Sreehari, 2012; Toro et al., 2019). Empirical evidence from diverse ESL settings underscores its efficacy in boosting motivation and communicative skills (Thamarana, 2015; Alsyed, 2018).

Yet, CLT’s adoption in EFL environments remains fraught with obstacles. Studies highlight cultural and institutional hurdles—such as exam-centric curricula, resource shortages, and entrenched views of teacher authority—that hinder communicative practices in East Asia (Li, 1998; Gorsuch, 2000; Hu, 2005; Jin and Yoo, 2019; Alharbi, 2022). Critically, these barriers reveal a tension between CLT’s learner-centered ethos and rigid, hierarchical educational traditions, often leading to superficial implementation rather than genuine pedagogical shift (Hu, 2005). Comparable issues persist in China, where high-stakes exams favor grammatical precision over fluency and interaction (Qi, 2007; Chen et al., 2016; Chen et al., 2024). Recent inquiries reveal deviations from CLT principles, driven by proficiency gaps and mismatched assessments (Chen et al., 2024; Zhu and Shu, 2017).

While extensive research examines in-service teachers’ CLT experiences (Doeur, 2022), pre-service teachers’ viewpoints are underexplored, despite their beliefs, attitudes, and perceptions forecasting future practices (Borg, 2003; Savignon and Wang, 2003). This gap is pivotal, as pre-service teachers embody the socialization of prevailing educational norms and foreshadow pedagogical evolution. In China, with an estimated 400 million English learners (British Council, 2023), how these educators conceptualize CLT holds profound implications for national policy and global language teaching. However, existing studies often rely on qualitative data, limiting generalizability, and overlook quantitative models examining attitudes as mediators in linking perceptions to beliefs (Borg, 2011; Sun et al., 2022).

The belief-practice gap further complicates this landscape: teachers endorse CLT theoretically but default to grammar-focused methods under systemic duress (Zhu and Shu, 2017; Yan, 2015). Probing this through pre-service lenses illuminates the negotiation of professional identities and orientations during formative phases, revealing how constraints mold future practices. Theoretically, Borg, Borg’s (2003, 2011) cognition framework posits that beliefs are shaped dynamically by perceptions of past experiences, yet attitudes—evaluative stances toward practices—serve as a crucial mediator, filtering perceptions into enduring beliefs. This mediation is underexplored quantitatively, particularly in pre-service EFL contexts, where attitudes could bridge experiential gaps and inform interventions (Sun et al., 2022). Emerging research on belief changes before and after practicums among Chinese pre-service teachers indicates that experiential interventions can shift cognitions toward more communicative orientations (Qiu et al., 2021).

This study probes how Chinese pre-service English teachers’ perceptions of secondary-level English teaching shape their attitudes and, subsequently, their beliefs, with attitudes mediating this relationship. It further explores how this interrelation varies by gender and English learning starting age, illuminating factors shaping CLT cognition. It addresses:

*RQ1*: What are pre-service English teachers’ perceptions of secondary school instructional practices?

*RQ2*: What are their attitudes toward grammar- versus communication-focused practices?

*RQ3*: What are their underlying beliefs about English learning?

*RQ4*: How do these interrelations differ by gender and English learning starting age?

Centering pre-service teachers, this research advances debates on CLT adaptability in EFL settings, bolsters teacher cognition with large-scale data, and informs reforms in China and analogous contexts. By examining the mediating role of attitudes and subgroup variations, it explores how targeted teacher training might bridge the belief-practice gap.

## 2 Literature review

This review synthesizes key developments in Communicative Language Teaching (CLT), its implementation challenges, and teacher cognition, with a focus on grammar- versus communication-focused orientations in EFL contexts. It highlights empirical gaps in pre-service teachers’ attitudes as mediators, setting the stage for this study’s quantitative mediation analysis.

### 2.1 Communicative Language Teaching and its principles

CLT, originating in the late 1970s, counters traditional grammar-translation methods by emphasizing communicative competence, integrating grammatical, sociolinguistic, discourse, and strategic elements (Richards and Rodgers, 2014; Larsen-Freeman and Anderson, 2011). Recent developments underscore hybrid models blending explicit grammar with interactive tasks like role plays to address EFL challenges (Sreehari, 2012; Toro et al., 2019; Dos Santos, 2020). Empirical studies in ESL settings affirm CLT’s efficacy in enhancing motivation and skills (Thamarana, 2015; Alsyed, 2018), while in China, research reveals tensions between grammar dominance and communicative goals (Chen et al., 2024). These findings highlight a need for quantitative examinations of pre-service cognitions in exam-oriented contexts like China to bridge theoretical endorsements with practical adaptations, which this study addresses through SEM modeling of perceptions, attitudes, and beliefs.

### 2.2 CLT in global implementation and challenges

Despite endorsement, CLT’s integration in EFL contexts faces systemic barriers, including exam-driven curricula and hierarchical teacher roles (Li, 1998; Gorsuch, 2000; Hu, 2005). In East Asia, educators struggle to reconcile CLT’s learner-centered approach with assessment traditions (Chung and Huang, 2009; Tootkaboni, 2019; Jin and Yoo, 2019). In China, high-stakes exams perpetuate grammar focus, leading to deviations from CLT principles due to proficiency gaps and class sizes (Qi, 2007; Yan, 2015; Chen et al., 2024; Chen et al., 2016). Global studies indicate hybrid implementations where theoretical support coexists with limited uptake (Karimi and Biria, 2017; Alharbi, 2022; Dang and Seals, 2018). Transitioning to teacher cognition, these challenges underscore the cognition-practice gap; this study fills empirical voids by quantifying how pre-service perceptions in high-stakes Chinese EFL settings influence attitudes and beliefs, informing localized reforms.

### 2.3 Teacher cognition and the cognition-practice gap

Teacher cognition encompasses beliefs, perceptions, and attitudes that shape instructional decisions, often rooted in early experiences and resistant to change (Borg, 2003, 2011). In CLT contexts, a cognition-practice gap emerges: educators affirm communicative principles but revert to grammar methods under institutional pressures (Savignon and Wang, 2003; Zhu and Shu, 2017). This gap is contextually mediated, with exam systems prioritizing accuracy over interaction (Qi, 2005; Chen et al., 2016). Recent quantitative studies on attitude-belief discrepancies in Chinese EFL teachers reveal implicit-explicit tensions (Sun et al., 2022; Gong et al., 2022). Attitudes, as evaluative stances, mediate this gap by filtering perceptions into beliefs, per Borg’s (2011) dynamic framework; yet, mediation models remain scarce in pre-service research. This study justifies and tests attitudes’ mediating role via SEM, addressing quantitative gaps in Chinese pre-service EFL cognition dynamics.

## 3 Materials and methods

This study employed a quantitative survey design to investigate pre-service English teachers’ perceptions, attitudes, and beliefs about CLT in Chinese secondary schools. Data were collected from a large sample to ensure statistical power for advanced analyses, including structural equation modeling (SEM). All procedures adhered to rigorous standards for reliability, validity, and replicability, aligning with guidelines for empirical research in applied linguistics (Plonsky and Derrick, 2016). The design allowed for subgroup analyses and robustness checks, enhancing the study’s methodological strength.

### 3.1 Participants

Participants were selected through a hybrid approach: convenience sampling from two accessible normal universities in China (key for training secondary English teachers), supplemented by snowball sampling (see section 3.3), with purposive screening applied post-collection to ensure alignment with the target population. These represent a subset of the broader population of pre-service English teachers (English-major undergraduates) across China. Participants comprised 865 English-major undergraduates at two normal universities in China—key institutions training future secondary school English teachers. The sample included 143 males (16.5%) and 722 females (83.5%), reflecting the gender imbalance typical in Chinese English education programs (Shan and Li, 2020). Regarding English learning onset, 67.4% began in primary school, 16.4% pre-primary, 15.4% in middle school, and 0.8% in high school. This distribution enabled subgroup analyses by gender and starting age. A priori power analysis using G*Power (Faul et al., 2009) confirmed the sample size provided > 0.95 power for detecting medium effects in SEM (Cohen’s *f*^2^ = 0.15, α = 0.05). The sample’s diversity in learning onset allowed exploration of how early exposure influences cognitions, a factor highlighted in recent studies on pre-service teacher development (Yang, 2019).

### 3.2 Instrument

The questionnaire, adapted from Savignon and Wang (2003) and contextualized for Chinese EFL, included 51 items across four sections: (1) demographics (e.g., gender, English starting age); (2) perceptions of secondary classroom practices (10 items: 5 grammar-focused, 5 communication-focused); (3) attitudes toward practices (10 items: parallel structure); and (4) beliefs about English learning (18 items: 10 grammar-focused, 8 communication-focused). Items used a 5-point Likert scale (1 = strongly disagree to 5 = strongly agree). To enhance validity and balance viewpoints, items were constructed with a mix of positive (e.g., endorsing communicative practices) and negative (e.g., favoring grammar dominance or critiquing interaction) statements across subscales, reducing acquiescence bias. Reverse-scored items were recorded during analysis (see section 3.4) to ensure consistent interpretation. Pilot testing was conducted with 50 similar but independent participants (English-major undergraduates from comparable institutions, not part of the main sample of 865) to ensure the instrument’s clarity and cultural appropriateness, yielding minor revisions. Bilingual back-translation ensured cultural equivalence (Brislin, 1970). Initial reliability checks showed acceptable Cronbach’s α (>0.70 for most subscales), with the grammar-perception subscale at 0.599. Despite this lower value, it was retained due to strong theoretical relevance to CLT contrasts in exam-oriented contexts, as item-total correlations (>0.30) indicated conceptual cohesion without redundant items. Sensitivity tests, excluding borderline items, confirmed consistent SEM results, supporting its inclusion without compromising overall validity. The instrument’s adaptation included items on digital tools to reflect contemporary EFL trends, enhancing relevance to current educational contexts (Guan et al., 2024).

### 3.3 Data collection

Data were gathered online via Wen Juan Xing (WJX), a secure platform compliant with data protection regulations. The questionnaire link was disseminated through university emails and social media, supplemented by snowball sampling for broader reach. Snowball sampling was used to extend the initial convenience sample, leveraging participants’ networks to increase diversity and response rate in an online setting where direct access to all potential respondents was limited. To mitigate biases, IP filtering eliminated duplicates, and attention-check items flagged invalid responses (e.g., straight-lining). From 1,107 initial submissions, 865 valid responses were retained based on criteria: completion of secondary education in China and current English-major enrollment. Collection occurred in early 2025, amid ongoing EFL reforms, enhancing timeliness. This timing coincided with increased AI adoption in education, potentially influencing responses on communicative practices (Gao et al., 2025).

### 3.4 Data analysis

Analyses were performed using SPSS 28.0 and AMOS 27.0, following a sequential protocol:

(1) Reliability: Cronbach’s α and item-total correlations assessed internal consistency.

(2) Exploratory Factor Analysis (EFA): On a random sample split (*n* = 432), principal axis factoring with oblique rotation extracted latent structures, assuming factor correlations.

(3) Confirmatory Factor Analysis (CFA): On the holdout sample (*n* = 433), model fit was evaluated via χ^2^/df (<3.0), CFI/TLI (>0.90), RMSEA/SRMR (<0.08) (Hu and Bentler, 1999). Convergent/discriminant validity used AVE and inter-construct correlations.

(4) SEM: A mediation model tested perceptions to attitudes to beliefs to address RQ3, emphasizing attitudes’ mediating role in linking past classroom experiences (perceptions) to future orientations (beliefs). Bootstrapping (5,000 resamples) was used for indirect effects to provide robust, non-parametric estimates, accounting for potential non-normality. Multi-group invariance assessed moderation by gender and starting age, examining whether the mediation paths varied across subgroups.

(5) Comparisons: *t*-tests/ANOVA explored subgroup differences.

(6) Robustness: Alternative models (e.g., reversed mediation) and Harman’s test checked for common method variance (<50% threshold). Missing data (<1%) were handled via FIML. Additional exploratory analyses examined correlations with learning onset, informed by recent cognition studies (Hsieh and Chuang, 2021). Additionally, to address potential response bias in Likert scales, reverse-scored items were included in subscales, and exploratory multicollinearity checks (VIF < 3.0) confirmed independence. These measures, aligned with applied linguistics standards (Plonsky and Derrick, 2016), mitigate cultural response tendencies in Chinese samples, ensuring mediation model’s validity for cross-cultural replication.

To ensure model robustness, assumptions for SEM were verified: multivariate normality via Mardia’s test (skewness < 3, kurtosis < 10), linearity through scatterplots, and homoscedasticity via Breusch-Pagan tests (all p > 0.05). Potential common method bias beyond Harman’s test was mitigated by procedural remedies, such as varied scale anchors and temporal separation in questionnaire sections. These steps align with best practices in applied linguistics (Plonsky and Derrick, 2016), facilitating replication. Future adaptations could incorporate longitudinal data to test causal inferences in the mediation model.

While SEM allows for testing mediation hypotheses, the cross-sectional design limits causal inferences, as temporal precedence among perceptions, attitudes, and beliefs cannot be established. This risks implying unwarranted causality; thus, results are interpreted as associations, with calls for longitudinal designs in future research (see Limitations in section 6.4) to validate directional paths.

### 3.5 Ethical considerations

Adhering to the Declaration of Helsinki and institutional protocols, participation was voluntary, anonymous, and withdrawable. No incentives were offered to avoid coercion, and data were stored securely with access limited to researchers. Potential biases (e.g., social desirability) were addressed through neutral item wording and anonymity assurances. Ethical considerations extended to digital data handling, ensuring compliance with data protection standards and enhancing participant confidentiality (Plonsky and Derrick, 2016).

## 4 Results

Descriptive and inferential statistics were computed to address the research questions, with SEM examining interrelationships. All analyses met assumptions (e.g., normality via skewness/kurtosis < |2.0|; multicollinearity VIF < 5.0). Effect sizes (Cohen’s *d* for *t*-tests; f for ANOVA) and 95% confidence intervals (CIs) are reported for key comparisons.

### 4.1 Reliability and validity of the instrument

Cronbach’s α ranged from 0.73 to 0.92 across subscales, indicating good internal consistency, except for grammar-focused perceptions (α = 0.599), retained after item analysis confirmed theoretical alignment; sensitivity tests excluding borderline items yielded consistent results. A random sample split enabled EFA (*n* = 432), extracting three factors (perceptions, attitudes, beliefs) explaining 68.4% variance (KMO = 0.87, Bartlett’s *p* < 0.001). CFA on the holdout (*n* = 433) showed strong fit: χ^2^/df = 2.54, CFI = 0.94, TLI = 0.93, RMSEA = 0.047 (90% CI: 0.042–0.052), SRMR = 0.039. Factor loadings > 0.60 (*p* < 0.001); AVE > 0.50 for convergent validity; square root of AVE > inter-correlations for discriminant validity (Fornell and Larcker, 1981).

The following are the Cronbach’s α coefficients for each subscale (Table 1).

**TABLE 1:** Cronbach’s α coefficients for each subscale.

Subscale	Cronbach’s α
Grammar-focused perceptions	0.599
Communication-focused perceptions	0.85
Grammar-focused attitudes	0.78
Communication-focused attitudes	0.82
Grammar-focused beliefs	0.73
Communication-focused beliefs	0.92

Finding: These results confirm the instrument’s overall reliability and validity, supporting the study’s objective to quantitatively model teacher cognition components (perceptions, attitudes, beliefs) for addressing all RQs.

### 4.2 Perceptions of secondary English classroom practices

Grammar-focused practices predominated (*M* = 3.74, SD = 0.54) over communication-focused ones [*M* = 3.23, SD = 0.88; paired *t*(864) = 18.76, *p* < 0.001, *d* = 0.64 (95% CI: 0.57–0.71)]. All grammar items exceeded the midpoint (3.0; one-sample *t*s > 10.0, *p*s < 0.001). Communication items like “emphasizing effective communication with occasional grammar” (*M* = 3.45) suggested partial presence (see Table 2). Exploratory correlations showed early starters perceiving more communicative practices (*r* = 0.15, *p* < 0.01).

**TABLE 2:** Means and standard deviations for perceptions of secondary English classroom practices.

Item type	Item example	Mean (M)	SD
Grammar-focused	Teachers provide explicit grammar explanations	3.85	0.62
Grammar-focused	Class focuses on grammar rules and translation	3.78	0.55
Grammar-focused	Use of grammar drills and tests	3.70	0.51
Grammar-focused	Emphasis on accuracy and error correction	3.72	0.53
Grammar-focused	Reading and writing based on grammatical structures	3.65	0.49
Grammar-focused overall	–	3.74	0.54
Communication-focused	Emphasizing effective communication with occasional grammar	3.45	0.92
Communication-focused	Establishing an environment for English use	3.38	0.89
Communication-focused	Using role plays and group discussions	3.15	0.85
Communication-focused	Focus on authentic interaction and meaning negotiation	3.20	0.87
Communication-focused	Encouraging students to communicate in English	3.00	0.84
Communication-focused overall	–	3.23	0.88

The following are the means and standard deviations for perceptions of secondary English classroom practices (Table 2).

Finding: Addressing RQ1, pre-service teachers perceive grammar-focused practices as dominant in secondary classrooms, highlighting the influence of exam-oriented systems on CLT cognition, consistent with the objective to explore perception-attitude-belief interrelations.

### 4.3 Attitudes toward classroom practices

Attitudes favored communication-focused practices (*M* = 3.82, SD = 0.69) over grammar-focused [*M* = 3.29, SD = 0.66; paired *t*(864) = 22.14, *p* < 0.001, *d* = 0.75 (95% CI: 0.68–0.82)]. Highest endorsement for “creating English-use environments” (*M* = 3.99; see Table 3), echoing pre-service shifts toward CLT amid reforms (Ye et al., 2025). Subgroup analysis indicated females’ stronger communicative attitudes correlated with higher beliefs (*r* = 0.22, *p* < 0.001).

**TABLE 3:** Means and standard deviations for attitudes toward classroom practices.

Item type	Item example	Mean (M)	SD
Grammar-focused	Teachers should provide explicit grammar explanations	3.40	0.70
Grammar-focused	Class should focus on grammar rules and translation	3.25	0.65
Grammar-focused	Using grammar drills and tests is effective	3.30	0.68
Grammar-focused	Emphasizing accuracy and error correction is important	3.28	0.64
Grammar-focused	Reading and writing should be based on grammatical structures	3.22	0.63
Grammar-focused overall	–	3.29	0.66
Communication-focused	Creating an environment for English use is ideal	3.99	0.72
Communication-focused	Emphasizing effective communication with occasional grammar	3.85	0.70
Communication-focused	Using role plays and group discussions is beneficial	3.80	0.68
Communication-focused	Focus should be on authentic interaction and meaning negotiation	3.78	0.67
Communication-focused	Encouraging students to communicate in English is key	3.68	0.66
Communication-focused overall	–	3.82	0.69

The following are the means and standard deviations for attitudes toward classroom practices (Table 3).

Finding: For RQ2, attitudes favor communicative practices despite grammar-dominant perceptions, supporting the objective of examining attitudes as mediators in CLT cognition.

### 4.4 Beliefs about English language learning

Beliefs leaned toward communication (*M* = 3.98, SD = 0.63) versus grammar [*M* = 3.66, SD = 0.60; paired *t*(864) = 15.92, *p* < 0.001, *d* = 0.54 (95% CI: 0.47–0.61)]. Strongest items included “formal grammar for proficiency” (*M* = 4.04) and “trial-and-error communication improves skills” (*M* = 4.12; see Table 4), indicating hybrid orientations consistent with evolving EFL cognitions. Early exposure positively correlated with communicative beliefs (*r* = 0.18, *p* < 0.001).

**TABLE 4:** Means and standard deviations for beliefs about English language learning.

Item type	Item example	Mean (M)	SD
Grammar-focused	Formal grammar study is essential for proficiency	4.04	0.65
Grammar-focused	Grammar rules are the foundation of language learning	3.75	0.62
Grammar-focused	Accuracy takes precedence over fluency	3.60	0.58
Grammar-focused	Translation exercises aid understanding	3.55	0.57
Grammar-focused	Error correction should be frequent	3.68	0.61
Grammar-focused	Grammar drills are effective	3.70	0.60
Grammar-focused	Focus on written language accuracy	3.62	0.59
Grammar-focused	Grammar knowledge promotes overall competence	3.65	0.58
Grammar-focused	Traditional methods are reliable	3.50	0.56
Grammar-focused	Exam-oriented grammar learning is necessary	3.55	0.57
Grammar-focused overall	–	3.66	0.60
Communication-focused	Engaging in trial-and-error communication improves skills	4.12	0.66
Communication-focused	Interaction is key to learning	4.05	0.64
Communication-focused	Authentic context use promotes fluency	3.95	0.62
Communication-focused	Meaning negotiation over form	3.98	0.63
Communication-focused	Role plays enhance practical ability	4.00	0.65
Communication-focused	Student-centered activities are effective	3.90	0.61
Communication-focused	Communicative environments foster motivation	4.02	0.64
Communication-focused	Strategic competence as important as grammar	3.85	0.60
Communication-focused overall	–	3.98	0.63

The following are the means and standard deviations for beliefs about English language learning (Table 4).

Finding: Responding to RQ3, beliefs lean toward communicative approaches with hybrid elements, aligning with the objective to explore belief formation through attitudes and perceptions.

### 4.5 Structural equation modeling

**FIGURE 1:**
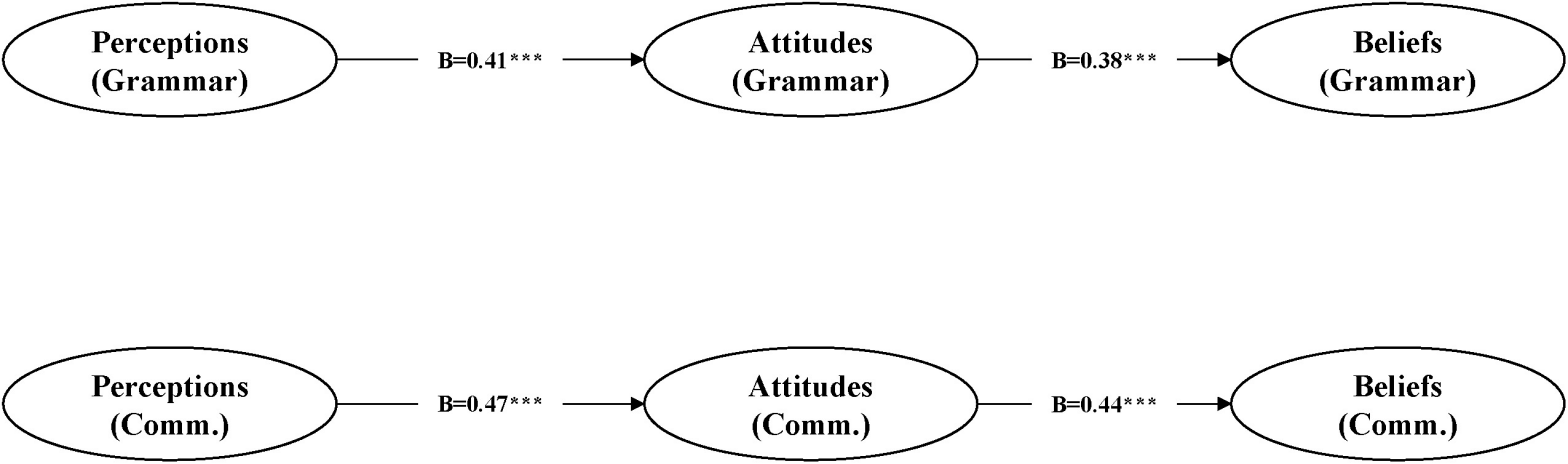
Structural equation model of pre-service teachers’ perceptions, attitudes, and beliefs. Standardized path coefficients are reported (***p < 0.001).

The mediation model fit well: χ^2^/df = 2.73, CFI = 0.95, TLI = 0.94, RMSEA = 0.049 (90% CI: 0.044–0.054), SRMR = 0.041. Perceptions predicted attitudes (grammar: β = 0.41, *p* < 0.001; communication: β = 0.47, *p* < 0.001). Attitudes predicted beliefs (grammar: β = 0.38, *p* < 0.001; communication: β = 0.44, *p* < 0.001). Attitudes mediated perceptions-beliefs, confirming the indirect effect [β = 0.19, bootstrapped 95% CI (0.12, 0.27), *p* < 0.001; see Figure 1].

The following details the SEM results, including path coefficients, standard errors, *t*-values, and *p*-values (Table 5).

**TABLE 5:** Structural equation modeling (SEM) results.

Path	β	SE	*t*	*p*-value
Perceptions (grammar) → Attitudes (grammar)	0.41	0.03	13.67	<0.001
Perceptions (comm.) → attitudes (comm.)	0.47	0.04	11.75	<0.001
Attitudes (grammar) → beliefs (grammar)	0.38	0.03	12.67	<0.001
Attitudes (comm.) → beliefs (comm.)	0.44	0.04	11.00	<0.001
Indirect effect (bootstrapped)	0.19	–	–	95% CI = (0.12, 0.27)

Finding: The SEM confirms attitudes’ mediating role in the perceptions-to-beliefs pathway (RQ3), supporting the objective to quantify interrelations in CLT cognition.

### 4.6 Group comparisons

Females endorsed communicative attitudes more strongly (*M* = 3.86) than males [*M* = 3.71; *t*(863) = 2.43, *p* = 0.015, *d* = 0.22 (95% CI: 0.08–0.36)]. No grammar differences (*p* > 0.05). Early starters (pre-primary) held stronger communicative beliefs (*M* = 4.05) than middle/high school starters [*M* = 3.89; *F*(2, 862) = 4.21, *p* = 0.016, η^2^ = 0.01 (95% CI: 0.00–0.02)], with *post hoc* Tukey confirming significance (*p* < 0.05). No other subgroup differences were significant. These variations underscore how demographic factors moderate the perceptions-attitudes-beliefs interplay in CLT cognition.

The following summarizes the group comparisons for communicative attitudes and beliefs (Table 6).

**TABLE 6:** Group comparisons for communicative attitudes and beliefs.

Group variable	Subgroup	Mean (communicative attitudes)	Mean (communicative beliefs)
Gender	Female	3.86	–
Gender	Male	3.71	–
Starting age	Pre-primary	–	4.05
Starting age	Primary	–	3.95
Starting age	Middle/high school	–	3.89

Finding: For RQ4, gender and starting age moderate communicative orientations, aligning with the objective to examine variations in cognition interrelations.

### 4.7 Robustness checks

Alternative models (reversed mediation: CFI = 0.88, RMSEA = 0.082; direct-only: CFI = 0.89, RMSEA = 0.079) fit poorly, supporting the hypothesized structure. Harman’s test (36% variance) ruled out substantial common method bias. Additional checks, including outlier removal, confirmed results’ stability.

The following compares model fit indices for robustness checks (Table 7).

**TABLE 7:** Model fit indices for robustness checks.

Model type	χ^2^/df	CFI	TLI	RMSEA	SRMR
Hypothesized (mediation)	2.73	0.95	0.94	0.049	0.041
Reversed mediation	–	0.88	–	0.082	–
Direct-only	–	0.89	–	0.079	–

Further robustness checks included sensitivity analyses excluding the low-reliability grammar-perception subscale (α = 0.599), yielding comparable SEM fit (χ^2^/df = 2.68, CFI = 0.94) and indirect effect [β = 0.18, 95% CI (0.11, 0.26)]. Subgroup interactions were tested via moderated mediation, revealing no significant gender moderation on the indirect path (index = 0.02, *p* = 0.45), but a marginal effect for early exposure (index = 0.05, *p* = 0.08), suggesting experiential factors warrant deeper exploration in larger samples.

Finding: Robustness confirms the model’s validity, supporting all RQs and the objective to reliably quantify mediation and variations in teacher cognition.

## 5 Discussion

This study elucidates the perceptions, attitudes, and beliefs of Chinese pre-service English teachers regarding grammar- and communication-focused instruction in secondary schools. Through a large-scale survey (*n* = 865) and SEM, the findings provide new insights into the interplay between perceptions, attitudes, and beliefs within a high-stakes EFL context. By quantifying the mediating role of attitudes, the study extends teacher cognition theory (Borg, 2011), demonstrating how evaluative stances during pre-service stages filter past experiences to shape future orientations. This section discusses the key results in depth, linking them to existing literature and exploring implications for practice, policy, and theory.

### 5.1 Prevalence of grammar-focused practices and enduring exam pressure

Consistent with prior research (Chen et al., 2016; Yan, 2015), participants reported that grammar-focused practices dominated their secondary school classrooms. This dominance is not merely a statistical artifact but reflects a deeper interplay of institutional inertia and cultural expectations, where the Gaokao’s emphasis on accuracy reinforces a “teaching to the test” paradigm that marginalizes communicative fluency (Qi, 2007). Although communicative activities were present to some degree, their secondary role suggests that systemic pressures—such as large class sizes and proficiency disparities—create a self-perpetuating cycle, limiting teachers’ agency to innovate (Chen et al., 2024; Zhu and Shu, 2017). Contextually, this aligns with exam-driven EFL challenges in East Asia, where hierarchical norms further entrench grammar as a proxy for educational rigor (Li, 1998; Gorsuch, 2000; Jin and Yoo, 2019). Theoretically, it illustrates how external constraints shape perceptual schemas early on, potentially foreclosing alternative pedagogies unless disrupted by reform.

### 5.2 Positive attitudes toward communicative approaches

Despite their grammar-oriented classroom experiences, pre-service teachers demonstrated stronger support for communicative approaches, especially the creation of environments conducive to English use. This attitudinal preference indicates a form of cognitive dissonance resolution, where exposure to global CLT ideals during university training fosters aspirational views that challenge lived realities, potentially seeding long-term pedagogical change (Savignon and Wang, 2003; Doeur, 2022). Subgroup findings, such as females’ stronger communicative attitudes, may stem from gendered socialization in Chinese education, where relational emphases align with CLT’s interactive ethos (Shan and Li, 2020; Ye et al., 2025). Explanatorily, this highlights attitudes as adaptive mechanisms, bridging the gap between exam-constrained pasts and reform-oriented futures, though implicit-explicit discrepancies underscore unresolved tensions (Sun et al., 2022).

### 5.3 Belief–practice gap and teacher cognition framework

The SEM results extend our understanding of this gap by showing that perceptions of prior classroom practices shaped pre-service teachers’ beliefs indirectly through attitudes. Subgroup variations further reveal moderated interrelations, with females and early starters showing stronger communicative alignments, suggesting experiential and sociocultural influences on cognition dynamics. This mediating effect suggests that beliefs are not direct reflections of past experiences but are filtered through evaluative stances developed during teacher education. Critically extending Borg (2003, 2011) framework, our model refines its dynamic cognition by empirically quantifying attitudes as a pivotal mediator, revealing how they actively reinterpret perceptions in context-specific ways, e.g., moderating exam pressures into hybrid beliefs. However, this extension invites critique: unmodeled moderators like gender and early exposure (as per subgroup effects) may oversimplify the framework in diverse EFL settings, echoing activity theory’s emphasis on sociocultural tools in cognition (Peng, 2024). For instance, while mediation aligns with Borg’s social mediation, it overlooks potential feedback loops where beliefs retroactively influence attitudes, warranting longitudinal refinement (Wang and Zhang, 2024; Kartchava et al., 2020). In China, this underscores cognition as negotiated amid Confucian authority norms, contrasting with less hierarchical contexts (Alharbi, 2022).

### 5.4 Cultural and policy influences

Beyond examinations, cultural traditions also shaped participants’ cognitions. The endorsement of statements such as “teachers should provide explicit grammar explanations” reflects the influence of Confucian heritage culture, which positions teachers as authoritative knowledge transmitters (Wei, 2016). This helps explain why grammar-focused beliefs coexist with positive communicative beliefs—a hybrid orientation shaped by both cultural expectations and global pedagogical trends. At the same time, policy reforms such as the New English Curriculum (NEC) promoted by China’s Ministry of Education (Fang and Garland, 2013) may explain the growing emphasis on communicative practices. Pre-service teachers’ strong support for communicative learning suggests that national policies are gradually reshaping pedagogical norms, even if classroom realities have yet to fully align. However, this shift may be uneven due to persistent cultural resistances, such as teacher-centered hierarchies, highlighting the need for policies that integrate local values with CLT adaptations rather than imposing external models.

### 5.5 Implications for teacher education and policy

The findings have several implications:

Teacher education: Programs should explicitly address the belief–practice gap by providing opportunities for pre-service teachers to critically reflect on their prior learning experiences and experiment with communicative methods during teaching practicums. Integrating AI tools can enhance this, as shown in studies on pre-service preparedness for AI-integrated education (Guan et al., 2024).

Curriculum and assessment: Sustainable CLT implementation requires alignment between classroom practices and assessment systems. Without examination reform, teachers’ willingness to adopt communicative methods will remain constrained.

Localized pedagogy: The coexistence of grammar- and communication-focused orientations suggests that CLT in China should evolve as a context-specific approach, integrating global principles with local realities, as seen in similar adaptations elsewhere (Ntirenganya, 2015).

### 5.6 Contributions to global EFL research

By focusing on pre-service teachers—a group underrepresented in CLT research—this study contributes to the broader teacher cognition literature. It provides empirical evidence that early learning experiences, attitudes, and beliefs interact in complex ways, shaped by both cultural heritage and policy reforms. These insights are particularly relevant to China but offer a cautious lens for other EFL contexts with similar exam and cultural pressures (e.g., Korea, Iran). The quantitative mediation model advances Borg’s framework, though its single-country, cross-sectional, self-report design limits generalizability; future studies should test it longitudinally and cross-culturally to refine CLT theory (Borg, 2015). Critically, while attitudes mediate in this context, sociodemographic moderators (e.g., gender) imply a need for intersectional approaches to disrupt grammar cycles equitably (Shan and Li, 2020; Wang and Zhang, 2024).

### 5.7 The role of AI integration in CLT

Although not the primary focus of this study, AI integration emerges as a promising tool to support CLT in exam-oriented contexts like China. AI can enhance communicative competence by providing personalized feedback, simulating real-world interactions (e.g., via chatbots for role plays), and reducing teacher workload through automated lesson planning and assessment (Guan et al., 2024). For pre-service teachers, AI fosters acceptance of communicative tasks by addressing proficiency gaps and offering equitable access to practice, potentially mitigating the belief-practice gap observed here (Gao et al., 2025; Liu et al., 2024). However, ethical concerns such as data privacy and digital divides must be addressed to ensure inclusive implementation, aligning with localized CLT reforms.

## 6 Conclusion

This study examined Chinese pre-service English teachers’ perceptions, attitudes, and beliefs regarding grammar- and communication-focused instruction in secondary school English classrooms. Drawing on a large-scale survey (*n* = 865) and structural equation modeling, the study revealed three key findings. Addressing RQ1 and the objective to explore foundational perceptions, participants perceived grammar-focused practices as dominant in their prior learning experiences, reflecting the influence of exam-oriented education. For RQ2, despite this background, they expressed stronger attitudinal support for communicative approaches, particularly the creation of interaction-rich environments. Third, the SEM analysis demonstrated that perceptions shaped beliefs indirectly through attitudes, underscoring the mediating role of attitudes in the teacher cognition process and fulfilling RQ3’s focus on interrelations. Fourth, subgroup variations by gender and starting age highlight moderated interrelations, responding to RQ4 and the objective to examine demographic influences on CLT cognition. These findings are enriched by comparisons with recent literature on implicit attitudes, belief dispositions, and cognition trajectories (Yi and Zhang, 2025; Sun et al., 2022; Wang and Zhang, 2024).

### 6.1 Theoretical contributions

The findings extend the literature on teacher cognition by highlighting the dynamic interplay between perceptions, attitudes, and beliefs during the formative stage of professional development (Borg, 2003, 2011). Specifically aligned with RQ3 and the mediation objective, the results refine our understanding of the belief–practice gap, showing that pre-service teachers’ beliefs are not direct replications of their prior experiences but are refracted through evaluative attitudes shaped by both teacher education and sociocultural contexts. This mediation mechanism contributes to ongoing debates about the contextual nature of teacher cognition and the adaptability of CLT in exam-driven EFL environments, integrating insights from intercultural competence studies (Gong et al., 2022). These conclusions are firmly supported by theories such as Borg’s dynamic cognition framework and activity theory (Peng, 2024), which emphasize mediated, context-sensitive processes; incorporating theories here is necessary to validate interpretations and highlight contributions beyond empirical data.

### 6.2 Practical implications

Several implications emerge for policy and practice: Teacher education programs should create structured opportunities for reflection and practice that challenge pre-service teachers to reconcile exam-oriented traditions with communicative pedagogy. Incorporating AI tools can facilitate this by targeting attitudes as mediators, as evidenced by studies on generative AI acceptance (Gao et al., 2025), where AI enables personalized simulations and feedback to enhance interactive learning. Curriculum and assessment reform is essential. Without alignment between communicative learning goals and high-stakes examinations, teachers’ willingness to implement CLT will remain constrained. Localized adaptation of CLT is necessary. Rather than treating CLT as a universal model, teacher educators and policymakers should acknowledge the hybrid orientation observed in China, where grammar-focused traditions coexist with growing communicative orientations. AI-mediated informal learning offers promising avenues for personalization (Liu et al., 2024). By leveraging attitudes as mediators and addressing subgroup variations (RQ4), policies could prioritize AI-driven simulations in curricula, fostering equitable shifts in cognition across EFL regions. supporting equitable shifts in cognition. By leveraging attitudes as mediators, policies could prioritize AI-driven simulations in curricula, fostering equitable shifts in cognition across EFL regions. This approach not only bridges gaps in China but offers scalable models for global adaptation, emphasizing empirical testing in comparative studies (Song et al., 2025).

### 6.3 Broader relevance

Although the study is situated in China, its findings resonate with other EFL contexts—such as Korea, Japan, Iran, and Saudi Arabia—where cultural traditions and exam pressures shape teachers’ pedagogical orientations. By centering pre-service teachers, this research highlights how future generations of educators may bridge the tension between global pedagogical trends and local educational realities. The incorporation of AI insights, particularly in mediating attitudes, extends relevance to digital-era EFL, addressing equitable education challenges.

### 6.4 Limitations and future directions

The study is not without limitations. First, reliance on self-reported survey data raises concerns about social desirability bias. Second, the focus on a single region in China may limit the generalizability of findings to other regions. Third, the cross-sectional design cannot capture the longitudinal evolution of teacher cognition.

Future research should therefore adopt mixed-method approaches, combining surveys with interviews and classroom observations to triangulate findings. Longitudinal studies tracking pre-service teachers into their in-service years would provide valuable insights into how attitudes and beliefs translate into practice over time. Additionally, comparative studies across multiple EFL contexts could deepen understanding of how cultural, institutional, and policy factors mediate the global-local dynamics of CLT, building on this study’s mediation and variation findings (RQ3 and RQ4). Exploring AI’s impact on cognition, such as in GenAI for teacher identity and corrective feedback, is a promising direction (Song et al., 2025; Kartchava et al., 2020).

To overcome these limitations, policymakers could fund hybrid training programs integrating AI simulations with practicum experiences, targeting attitude mediation to accelerate CLT shifts. Comparative research in diverse EFL regions (e.g., Vietnam or Iran) could validate the model’s cross-cultural applicability, refining global standards for teacher cognition amid technological advancements (Borg, 2015; Song et al., 2025).

## Data Availability

The original contributions presented in this study are included in this article/supplementary material, further inquiries can be directed to the corresponding author.
